# The moderating effect of perceived organizational support: The impact of psychological capital and bidirectional work-family nexuses on psychological wellbeing in tourism

**DOI:** 10.3389/fpsyg.2023.1064632

**Published:** 2023-02-23

**Authors:** Na Bai, Zhen Yan, Rosly Othman

**Affiliations:** ^1^Graduate School of Business, Universiti Sains Malaysia, Penang, Malaysia; ^2^School of Economics and Management, Wenshan University, Wenshan, China; ^3^School of Hotel Management, Qingdao Vocational and Technical College of Hotel Management, Qingdao, China

**Keywords:** perceived organizational support, bidirectional work-family nexuses, psychological capital, psychological wellbeing, tourism

## Abstract

The novel coronavirus (COVID-19) has inflicted unprecedented damage on the tourism industry. However, the psychological health fallout of COVID-19 on tour guides has not received empirical attention yet. Therefore, the present study aims to examine how psychological capital (PsyCap) improve tour guides’ psychological wellbeing (PWB), the mediating effects of work-family conflict (WFC), family-work conflict (FWC), work-family facilitation (WFF) and family-work facilitation (FWF), and the moderating effect of perceived organizational support (POS). For this quantitative research, the data were collected from 276 tour guides in China. The results indicate that PsyCap significantly mitigates two directions of work-family conflict and intensifies two directions of work-family facilitation in order to promote tour guides’ PWB. Furthermore, POS moderates the direct effects of two directions of conflict and facilitation on PWB and also moderates the indirect effects of PsyCap on the aforesaid outcome *via* two directions of conflict and facilitation. Theoretical and practical implications, limitations and future research directions are provided.

## 1. Introduction

COVID-19 outbreak has caused unprecedented economic losses for the fragile tourism industry (Buheji et al., 2020). The highly infectious novel coronavirus continues to thwart the development of the sector and seriously affect the work and family life of practitioners (Kaushal and Srivastava, 2021). Although the pandemic in China has been effectively controlled and tourism industry is slowly recovering (Czerny et al., 2021), however, how to improve the psychological wellbeing (PWB) of tour guides is still worth great attention in the post-epidemic era. Psychological capital (PsyCap) is a significant resource foundation for individuals and a positive psychological status characterized by self efficacy, optimism, hope and resilience (Luthans et al., 2007a) Prior studies showed that PsyCap could significantly influence employees’ work behaviors and wellbeing (Rabenu et al., 2017; Darvishmotevali and Ali, 2020). Thus, tourism agencies should focus on promoting employees’ PsyCap so as to positively affect their PWB.

Tourism itself is a highly stressful industry, as it is labor-intensive and involves frequent rotations, work overload, irregular working hours, and long time being away from home (Luoh and Tsaur, 2014). The COVID-19 outbreak has made the stressful and difficult conditions of tourism industry employees even worse (Li et al., 2022). Therefore, tour guides tend to experience more work-family conflict (WFC) in such situation (Yorulmaz and Sevinc, 2021). Grzywacz (2000) highlighted that in the process of extensive studying about WFC, some researchers have begun to wonder whether there are any potential benefits for the linkage between work and family life. Finally, a new concept explaining the positive interaction between work and family was developed and identified as work-family facilitation (WFF) (Voydanoff, 2004). Then, researchers found that work-family conflict could be generated by bidirectional effects, respectively, work-family conflict (WFC) and family-work conflict (FWC) (Higgins et al., 1992). In the same way as the concept of work-family conflict, facilitation should also be understood as bidirectional phenomenon, namely, work-family facilitation (WFF) and family-work facilitation (FWF). Despite many psychological determinants have been verified to be associated with WFC or WFF (Allen and Kiburz, 2012; Obrenovic et al., 2020; Zhu et al., 2021), less is known about how PsyCap alleviates two directions work-family conflict and promotes two directions of work-family facilitation simultaneously especially in tourism industry. Karatepe and Karadas (2014) have investigated the impact of PsyCap on the conflicts of the work-family interface in the context of hospitality industry in Romania. In addition, Yan et al. (2022) have confirmed that PsyCap could significantly alleviate two directions of WFC and promote flight attendants’ job performance in Malaysia, however, relevant research is still lacking in China.

García-Cabrera et al. (2018) found evidence of the impact of perceived supervisor support on WFC and called for other forms of work support, such as co-worker support and perceived organizational support (POS). However, to our best knowledge, there are few studies which examined the association between POS and WFC and WFF in tourism sector. Some studies have demonstrated that organizational support and flexible work arrangements are useful in mitigating WFC and promoting WFF so as to enhance job satisfaction and wellbeing (Wattoo et al., 2018). Such research in China is still lacking. Thus, the main objective of this study is to analyze the impact of PsyCap on WFC, FWC, WFF, FWF, and PWB. We also explored the mediating role of two directions of conflict and facilitation in the relationship between PsyCap and PWB, while also examining whether POS moderates these relationships.

## 2. Literature review

According to conservation of resources (COR) theory of Hobfoll (1989), individuals desire to acquire, preserve and maintain their limited resources. These resources are specifically divided into material resources, conditional resources, personality traits, and energy resources. PsyCap is a type of personality resource (Avey et al., 2010a) and the work-family nexuses discussed before are a summary of employees’ social relations, which belong to conditional resources. Apparently, WFC or FWC is a state of conditional resource loss. Individuals who suffer a loss of resources are likely not to feel PWB. On the contrary, when employees experience WFF or FWF, they will acquire some skills because of their work or family, which is conducive to their PWB. What’s more, POS is also a valuable resource that enhances employees’ confidence in coping with the demands of their role (Jawahar et al., 2007). A high level of POS provides employees with abundant resources to better deal with conflicts and facilitation in the work-family domain (Wattoo et al., 2018). On the contrary, employees with low level of POS is difficult to achieve PWB (Mansouri et al., 2022).

### 2.1. Psychological capital and psychological wellbeing

Luthans et al. (2007a) defined PsyCap as “an individual’s positive psychological state of development” which is a multidimensional construct identified by four positive psychological resources, respectively, hope, optimism, self-efficacy and resilience. PWB refers to individual’s judgments based on the frequency or intensity of positive psychological states (Diener et al., 1985). Employees’ PWB is closely associated with the overall effectiveness of employees’ psychological functioning (Kim and Jang, 2022). Thus, it is logical to think that PsyCap may be related to PWB. Researchers in health psychology have demonstrated that PWB is influenced by: hope (Satici, 2016; Pleeging et al., 2021), resilience (Mak et al., 2011; Prime et al., 2020), self-efficacy (Karademas, 2006; Marshall et al., 2020), and optimism (Scheier and Carver, 1992; Karademas, 2006). What’s more, a positive relationship has been found between PsyCap and wellbeing in previous research (Siu et al., 2015; Roemer and Harris, 2018).

Avey et al. (2010a) highlighted that COR theory has been employed to attribute how PsyCap relates to employees’ wellbeing, since employees cognitively assess stressful situations and positively adapt by maintaining resources. Based on COR theory, an individual’s ability to acquire and maintain resources is both a means and an end (Lazarus and Folkman, 1984). A means refers to a method to achieve goals and success and an end includes adaptation, coping, and wellbeing, depending on individual’s cognitive evaluation. Personal resources like PsyCap facilitate the accurate evaluation of the current situation. The positive cognitive and behavioral processes of PsyCap are a significant predictor of wellbeing (Avey et al., 2010b). Individuals with high levels of PsyCap are able to interpret situations in a positive and helpful manner, feel energized and motivated, and have the ability to show adaption in the face of adversity (Roemer and Harris, 2018; Yan et al., 2021b). In addition, Avey et al. (2010b) have analyzed the relationship between employees’ level of PsyCap and two measures of PWB (Indexes of Psychological Wellbeing and General Health Questionnaire). The results demonstrated that PsyCap could explain additional variance in these two wellbeing measures over time. Thus, we hypothesized:

**H1:** PsyCap is positively related to PWB.

### 2.2. The mediating role of two directions of conflict and facilitation

According to conservation of resources theory, to prevent resource loss and to enhance resource gain, individuals invest in resources that are utilized to meet role demands and gain PWB (Hobfoll, 2002). When resources are insufficient, individuals will strive to maintain their own resources in order to effectively cope with the situation (Hobfoll, 1989). On the contrary, the accumulation of resources allows individuals to reinvest in their jobs. When employees have sufficient resources and their investments can get good returns, they tend to invest “redundant” resources to obtain additional resources.

On the one hand, when employees are in a state of lack of resources, they will take actions to reduce their “surplus” and their further loss of resources (Hobfoll, 1989). Those employees facing conflict between the work and family domains believe that they do not have adequate resources to pursue PWB. To be specific, the challenging characteristics of high level of PWB put forward higher requirements for employees’ resources investment. In this case, in order to preserve their surplus resources, employees reduce their work input, which may lead to a decline in the level PWB. On the other hand, individuals with more resources can use their existing resources to obtain even more resources, and this will encourage them to have a positive psychological state and work behavior (Hobfoll et al., 2018). Facilitation from family and work domains makes employees believe that they are in a state of sufficient resources, which in turn makes them more inclined to invest “redundant” resources (including time, energy, etc.) to pursue success and PWB.

The conservation of resources theory posits that employees who possess high PsyCap can deal with problems arising from both work and family domains (Karatepe and Karadas, 2014), which in turn might lead to higher PWB (Siu, 2013). First, Hobfoll (2002) believed that employees with key resources such as resilience or self-efficacy tended to be more able to select, change and implement their other resources to meet stressful demands. To be specific, such employees can invest their personal resources in order to be capable of managing WFC or FWC and recover from losses. As a result, they can establish a balance between their work (family) and family (work) roles. Conversely, if they don’t have sufficient personal resources, they will not be able to overcome such difficulties in the work-family interface. Second, individuals with higher PsyCap have a tendency to be more successful, adapt better and achieve high levels of PWB (Avey et al., 2011).

Unlike considerable research on work-family conflict, few studies have reported on the relationship between work-family facilitation and its actual consequences (Wu and Chang, 2020). Limited evidence has been suggested that work-family facilitation had a positive impact on job outcomes (Choi and Kim, 2012). Accordingly, this study proposed the following hypotheses:

**H2a:** WFC mediates the relationship between PsyCap and PWB.

**H2b:** FWC mediates the relationship between PsyCap and PWB.

**H2c:** WFF mediates the relationship between PsyCap and PWB.

**H2d:** FWF mediates the relationship between PsyCap and PWB.

### 2.3. The moderating role of perceived organizational support

Scholars have attempted to understand the possible antecedents or moderators that affect two directions of conflict and facilitation in recent decades (e.g., Karatepe and Bekteshi, 2008; Choi and Kim, 2012; Zhao and Ghiselli, 2016). A construct that has been widely utilized to determine whether an organization is generally supportive is POS. Eisenberger et al. (1986), in their definition of POS, suggested that employees develop a general perception about the degree to which organizations value their contributions and care about their wellbeing. POS is also regarded as assurance that aid will be available from the organization when it is needed to deal with stressful situations (Rhoades and Eisenberger, 2002). Consistent with the COR theory (Hobfoll, 1989), POS is a type of valued resource that bolsters employee confidence in coping with role demands (Jawahar et al., 2007). High level of POS provides employees with abundant resources, better dealing with conflicts and advancing facilitation in work-family domains (Wattoo et al., 2018). Without such resources, a low POS context, employees’ PWB is hard to achieve.

Although much is now known about the antecedents and consequences of two directions of conflict and facilitation, relatively little research has focused on the role of POS in work-family domains. Several scholars have argued that POS has significant relationships with work-family conflict or facilitation (e.g., Selvarajan et al., 2016; Wattoo et al., 2018; Zheng and Wu, 2018). Thus, we hypothesized:

**H3a:** POS moderates the mediating role of WFC between PsyCap and PWB.

**H3b:** POS moderates the mediating role of FWC between PsyCap and PWB.

**H3c:** POS moderates the mediating role of WFF between PsyCap and PWB.

**H3d:** POS moderates the mediating role of FWF between PsyCap and PWB.

### 2.4. Research model

**FIGURE 1:**
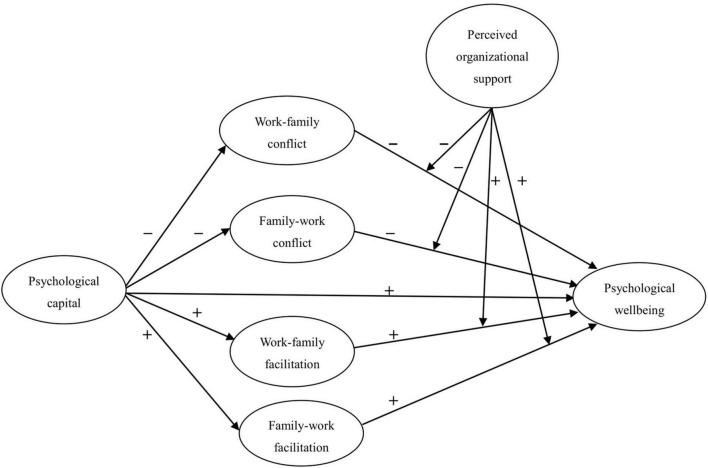
Conceptual model for the analysis.

The research model shown in Figure 1 demonstrates the hypothesized relationships. Specifically, the model aims to examine how PsyCap influences two directions of conflict and facilitation (including WFC, FWC, WFF, and FWF) and PWB. In addition, the mediating effects of two directions of conflict and facilitation, and the moderating effect of POS will also be tested.

## 3. Materials and methods

### 3.1. Procedure and participants

We used convenience sampling technique to collect data from travel agencies in the provinces of Yunnan and Sichuan, from October 2021 to December 2021. With the support of managers of the travel agencies, online survey link was delivered to every potential participant. Target respondents of the research were full-time tour guides since they have a better overall understanding and experience with the conflict and facilitation between work and family than part-time tour guides. The self-administered questionnaires consisted of information about the assurance of confidentiality (Podsakoff et al., 2003). Those who completed the survey were given a small gift to encourage their participation. Finally, 322 questionnaires were returned. After eliminating the invalid questionnaires with missing answers and too many same options, 276 valid questionnaires were obtained with a response rate of 83.1%. The composition of the valid samples is shown in Table 1. In terms of gender, 69.2% of the participants were female; 51.5% were aged 18–30; 54.7% were unmarried; 66.6% held a junior college degree or above; and 36.6% has worked for 1–3 years.

**TABLE 1:** The characteristics of the respondents (*n* = 276).

Demographic variables	Frequency	(%)
**Gender**		
Male	85	30.8%
Female	191	69.2%
**Age**		
18–30 years	142	51.5%
31–40 years	108	39.1%
41 years and above	26	9.4%
**Marriage**		
Unmarried	151	54.7%
Married	125	45.3%
**Education**		
Junior high	28	10.2%
Senior high	64	23.2%
Junior college	142	51.4%
College	42	15.2%
**Tenure**		
Less than 1 year	59	21.4%
1–3 years	101	36.6%
4–6 years	64	23.2%
6 years above	52	18.8%

### 3.2. Measures

In the current study, we selected mature scales available both at home and abroad, which have been widely utilized in tourism and hospitality industry to ensure the reliability and effectiveness of the measurement variables. We translated these items from English into Chinese in accordance with the “translation and back translation” procedure (Brislin, 1980). Based on the following five measurement tools, totally 52 items, were included in the initial model.

#### 3.2.1. PsyCap

We used a 24-item scale proposed by Luthans et al. (2007a) to measure PsyCap. This scale consists of four dimensions and responses are recorded on a Likert six-point scale ranging from 1 (strongly disagree) to 6 (strongly agree). A sample item is “I can get through difficult times at work because I’ve experienced difficulty before.” The Cronbach’s α coefficient for the four dimensions were 0.888, 0.863, 0.865, and 0.839, respectively.

#### 3.2.2. Work-family conflict and facilitation

WFC, FWC, WFF, and FWF were measured using 16 items from Grzywacz and Marks (2000). Specifically, four items each from this scale were used to measure the two types of conflict and the two types of facilitation. 5-point Likert scales ranging from 1 (never) to 5 (all the time) were adopted. The four sampling items corresponding to four variables are “Stress at work makes you irritable at home.” “Responsibilities at home reduce the effort you can devote to your job.” “The skills you use on your job are useful for things you have to do at home.” “Talking with someone at home helps you deal with problems at work.” The Cronbach’s α coefficient for the four variables were 0.866, 0.863, 0.866, and 0.870.

#### 3.2.3. Psychological wellbeing

We utilized a 6-item short version of the Psychological General Wellbeing index (PGWB-S) developed by Grossi et al. (2006). The response format was a 5-point Likert scale ranging from 1 (almost never) to 5 (very often). A sample item is “I felt tired, worn out, used up, or exhausted during the past month.” The Cronbach’s α coefficient for this scale was 0.811.

#### 3.2.4. Perceived organizational support

A six-item scale taken from Eisenberger et al. (1986) was utilized to assess POS. The items were rated on a five-point scale ranging from 1 (strongly disagree) to 5 (strongly agree). A sample item is “This organization is willing to help me when I need a special favor.” The Cronbach’s α coefficient for this scale was 0.885.

#### 3.2.5. Control variables

Considering that demographic variables may affect tour guides’ PWB, we controlled gender, age, marriage, education and tenure.

### 3.3. Data analytic strategy

The SPSS 23.0 was utilized for descriptive statistics of respondents’ demographic information and to analyze the correlations among all variables. Confirmatory factor analysis (CFA) was performed to appraise the measurement model and provide evidence of validity and reliability with AMOS 23.0 (Fornell and Larcker, 1981). The hypotheses were examined with AMOS 23.0 and PROCESS (version 3.2) (Anderson and Gerbing, 1988; Hayes, 2017), which have been widely adopted in tourism industry (Tu et al., 2020; Li et al., 2021). These analyses were performed based on bootstrapping methods (Hayes, 2017), which can provide reliable estimation of the indirect effects (Preacher and Hayes, 2008). Bootstrapping techniques utilize 95% confidence interval to verify significant results, that is, a 95% confidence interval excluding zero indicates that the mediating or moderating effect is statistically significant.

## 4. Results

### 4.1. Common method bias test

Common method variance (CMV) refers to the artificial co-variation among independent and dependent variables caused by use of the same subjects or data sources, similar measurement or project characteristics (Podsakoff et al., 2003). In this study, Harman’s single-factor test was conducted to test the degree of variation of the sample data as recommended by Podsakoff et al. (2003). The result that the cumulative percent of first factor was 27.443% (less than the critical value of 40%) implied that no single factor was apparent in the un-rotated factor structure, which indicated that common method bias was not a serious issue in the current study.

### 4.2. Full measurement model validation

Research on structural equation modeling usually contains multiple latent constructs, each of which contains several or even dozens of indicators. As the number of indicators increases, the complexity of the model and the required sample size also increase. In many cases, it is not easy to collect sample and the model is too complex to establish the relationship between latent constructs. Therefore, in current study, item parceling technique was utilized to test PsyCap as a second-order latent construct. Item parceling has obvious advantages which include higher reliability (Kishton and Widaman, 1994), high degree of commonality (Little et al., 2002), closer to normal data distribution pattern (Bagozzi and Heatherton, 1994), and more convergent (Marsh et al., 1998) and better model fit (William et al., 2004).

After an inspection of the CFA, the results revealed that it was necessary to delete four low factor loading items. The revised full measurement model presented a satisfactory fit with the data. To be specific, all standardized factor loadings range from 0.568 to 0.887, which meant that all items could measure their corresponding constructs effectively. What’s more, all the constructs’ composite reliability is larger than the minimum threshold of 0.7. Furthermore, the latent variables’ AVE values were larger than the threshold value of 0.50. That said, Larcker (1981) asserted that an AVE value above 0.36 could also comprise a reluctantly accepted standard. In brief, the above findings also indicated that all the indicators measuring constructs were one-dimensional in the full measurement model. Fit indices strongly supported the hypothesized full measurement model, for instance, *X*^2^ = 700.785, df = 442; Normed *X*^2^ = 1.585; CFI = 0.947; TLI = 0.941; IFI = 0.948; NFI = 0.870; RMSEA = 0.046; and AIC = 936.785.

In this research, discriminant validity was evaluated *via* the following method. In brief, if all the values of AVE were larger than the corresponding squared correlation coefficients, the discriminant validity of the measurement model was achieved. This method was deemed as the most rigorous test of discriminant validity. As shown in Table 2, all constructs’ squared correlation coefficients are smaller than the corresponding AVE values, thus a strong proof of the discriminant validity for all constructs was provided.

**TABLE 2:** Means, standard deviations, correlations, and discriminant validity.

Variables	Mean	SD	1	2	3	4	5	6	7
1. PsyCap	3.680	0.813	***0***.***566***						
2. WFC	3.554	1.028	-0.333***	***0***.***620***					
3. FWC	3.563	1.090	-0.423***	0.259***	***0***.***616***				
4. WFF	3.526	1.070	0.487***	-0.199**	-0.195**	***0***.***620***			
5. FWF	3.625	1.026	0.296***	-0.248***	-0.241***	0.290***	***0***.***632***		
6. PWB	3.707	0.912	0.554***	-0.425***	-0.410***	0.430***	0.415***	***0***.***556***	
7. POS	3.402	0.644	0.433***	-0.403***	-0.324***	0.347***	0.451***	0.443***	***0***.***529***

**P < 0.005, ***P < 0.001.

The square root of AVE for each variable is shown with bold italics.

### 4.3. Structural model estimation and hypotheses testing

As the reliability and validity of full measurement model had been verified, the structural model was going to be evaluated and the hypotheses were going to be tested in the next step. After statistical analysis, fit indices from the following structural model demonstrated that the proposed structural model was satisfactory.

#### 4.3.1. Direct effects and hypotheses testing

Standardized parameter estimates, *t*-values and *p*-values for the structural model were calculated as a part of path analysis in AMOS. According to the results showed in Table 3, all direct paths in the structural model were statistically significant. PsyCap had a positive direct effect on PWB (β = 0.281; *p* = 0.001). Thus, H1 was supported. In addition, PsyCap negatively influenced two directions of work-family conflict (β =−0.355, *p* < 0.001; β =−0.433, *p* < 0.001) and positively influenced two directions of work-family facilitation (β = 0.496, *p* < 0.001; β = 0.329, *p* < 0.001). Finally, WFC and FWC had negative impact on PWB (β =−0.210, *p* < 0.001; β =−0.164, *p* = 0.011) while WFF and FWF had positive impact on PWB (β = 0.170, *p* = 0.012; β = 0.190, *p* = 0.001).

**TABLE 3:** Results for hypotheses tests.

Path	Relation	Std. parameter estimates	*t*-value	*p*
PsyCap—>PWB	Positive	0.281	3.256	0.001
PsyCap—>WFC	Negative	-0.355	-4.958	***
PsyCap—>FWC	Negative	-0.433	-5.874	***
PsyCap—>WFF	Positive	0.496	6.566	***
PsyCap—>FWF	Positive	0.329	4.630	***
WFC—>PWB	Negative	-0.210	-3.380	***
FWC—>PWB	Negative	-0.164	-2.546	0.011
WFF—>PWB	Positive	0.170	2.506	0.012
FWF—>PWB	Positive	0.197	3.245	0.001

***P < 0.001.

PsyCap, psychological capital; PWB, psychological wellbeing; WFC, work-family conflict; FWC, family-work conflict; WFF, work-family facilitation; FWF, family-work facilitation.

#### 4.3.2. Mediating effects and hypotheses testing

As can be seen in Table 4, to identify the mediating role of two directions of conflict and facilitation, bias-corrected and percentile bootstrapping methods were utilized (Baron and Kenny, 1986). If the bootstrapped CI does not include zero, it means that the mediating effect differs from zero. In this research, 95% bias-corrected CI was estimated with 2000 bootstrapped samples for two methods. To summarize, both the bias-corrected and percentile bootstrapping methods showed that two directions of conflict and facilitation could mediate the relationship between PsyCap and PWB, which supported H2a, H2b, H2c, and H2d.

**TABLE 4:** Results for mediating effects.

			Bias-corrected 95% CI		Percentile 95% CI		
Effects	Path	β	LLCI	ULCI	LLCI	ULCI	*p*
Total Effects	PsyCap–>PWB	0.575	0.401	0.708	0.411	0.715	0.001
Indirect Effects	PsyCap->WFC->PWB	0.074	0.026	0.142	0.02	0.13	0.001
	PsyCap->FWC->PWB	0.071	0.013	0.135	0.013	0.135	0.011
	PsyCap->WFF ->PWB	0.084	0.019	0.168	0.018	0.163	0.009
	PsyCap->FWF->PWB	0.065	0.027	0.129	0.022	0.120	0.004
Direct Effects	PsyCap–>PWB	0.281	0.049	0.465	0.063	0.475	0.017

PsyCap, psychological capital; PWB, psychological wellbeing; WFC, work-family conflict; FWC, family-work conflict; WFF, work-family facilitation; FWF, family-work facilitation.

#### 4.3.3. Moderating effects and hypotheses testing

**FIGURE 2:**
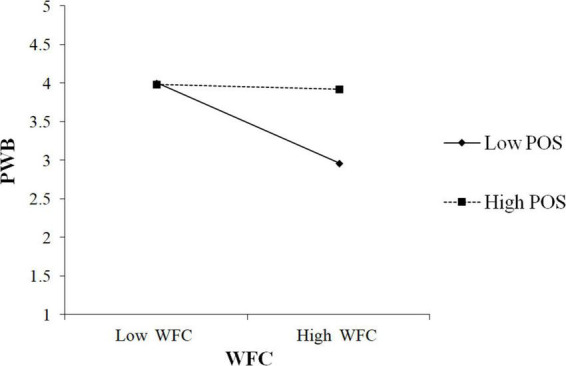
Effects of work-family conflict (WFC) and perceived organizational support (POS) on psychological wellbeing (PWB).

In order to avoid multicollinearity, mean-centered two directions of conflict and facilitation and POS were adopted before generating the interaction between them. As shown in Table 5, the interaction of WFC and POS was positively associated with PWB (β = 0.28, *p* < 0.001). To better understand the moderating effect, we followed Aiken and West (1991) to plot the interaction by calculating slopes one SD (standard deviation) above and one SD below the average value of POS. Figure 2 showed the interaction pattern. The findings in Table 5 also revealed that the conditional indirect effect was stronger in the low POS condition (β = 0.14, CI 0.07, 0.23), and weaker in the high condition (β = 0.02, CI –0.02, 0.07). In addition, the moderated mediation index denoted the difference between high and low conditional effects (Index =−0.09, CI –0.17, –0.03) and the CI did not include zero. Thus, these results supported H3a.

**TABLE 5:** Results of moderated-mediation of WFC.

	WFC (M)			PWB (Y)		
Variables	β	SE	*p*	β	SE	p
Gender	-0.16	0.13	0.22	0.07	0.10	0.49
Age	0.03	0.11	0.77	-0.07	0.08	0.35
Marriage	0.08	0.14	0.59	-0.12	0.11	0.27
Education	0.08	0.08	0.28	0.09	0.06	0.12
Tenure	0.00	0.07	0.97	0.02	0.06	0.78
PC (X)	-0.33	0.08	0.00***	0.32	0.07	0.00***
POS (W)				0.22	0.08	0.005**
WFC (M)				-0.23	0.05	0.00***
M*W				0.28	0.07	0.00***
	*R*^2^ = 0.24			*R*^2^ = 0.36		
**POS**			**B**	**SE**	**LLCI**	**UICI**
–1 SD			0.14	0.04	0.07	0.23
M			0.07	0.02	0.03	0.12
+1 SD			0.02	0.02	-0.02	0.07
Index			-0.09	0.03	-0.17	-0.03

**P < 0.005, ***P < 0.001.

X, independent variable; W, moderator; M, mediator; Y, dependent variable; B, unstandardized coefficient; SE, standard error; P, probability; SD, standard deviation; LLCI, lower-level confidence interval; ULCI, upper-level confidence interval.

**FIGURE 3:**
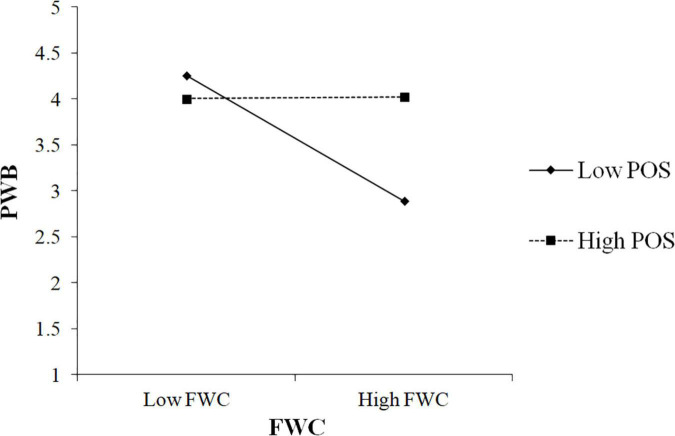
Effects of family-work conflict (FWC) and POS on PWB.

As shown in Table 6, the interaction of FWC and POS was positively associated with PWB (β = 0.43, *p* < 0.001). To better understand the moderating effect, we followed Aiken and West (1991) to plot the interaction by calculating slopes one SD (standard deviation) above and one SD below the average value of POS. Figure 3 showed the interaction pattern. The findings in Table 6 also revealed that the conditional indirect effect was stronger in the low POS condition (β = 0.25, CI 0.15, 0.35), and weaker in the high condition (β = –0.04, CI –0.10, 0.02). In addition, the moderated mediation index denoted the difference between high and low conditional effects (Index =−0.22, CI –0.30, –0.13) and the CI did not include zero. Thus, these results supported H3b.

**TABLE 6:** Results of moderated-mediation of FWC.

	FWC (M)			PWB (Y)		
Variables	β	SE	*p*	β	SE	*p*
Gender	0.16	0.13	0.23	0.09	0.10	0.35
Age	-0.06	0.11	0.61	-0.09	0.08	0.26
Marriage	0.24	0.14	0.10	-0.12	0.10	0.23
Education	-0.10	0.08	0.19	0.08	0.06	0.17
Tenure	0.11	0.07	0.16	0.00	0.05	0.88
PC (X)	-0.51	0.08	0.00***	0.26	0.07	0.00***
POS (W)				0.24	0.08	0.001**
FWC (M)				-0.18	0.04	0.00***
M*W				0.43	0.06	0.00***
	*R*^2^ = 0.16			*R*^2^ = 0.40		
**POS**			**B**	**SE**	**LLCI**	**UICI**
-1 SD			0.25	0.05	0.15	0.35
M			0.07	0.03	0.02	0.12
+1 SD			-0.04	0.03	-0.10	0.02
Index			-0.22	0.04	-0.30	-0.13

**P < 0.005, ***P < 0.001.

X, independent variable; W, moderator; M, mediator; Y, dependent variable; B, unstandardized coefficient; SE, standard error; P, probability; SD, standard deviation; LLCI, lower-level confidence interval; ULCI, upper-level confidence interval.

**FIGURE 4:**
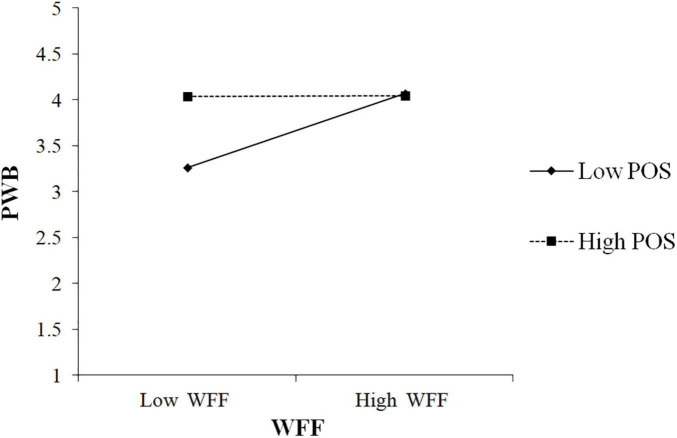
Effects of work-family facilitation (WFF) and POS on PWB.

Table 7 showed that the interaction of WFF and POS was negatively associated with PWB (β =−0.20, *p* = 0.002). To better understand the moderating effect, we followed Aiken and West (1991) to plot the interaction by calculating slopes one SD (standard deviation) above and one SD below the average value of POS. Figure 4 showed the interaction pattern. The findings in Table 7 also revealed that the conditional indirect effect was stronger in the low POS condition (β = 0.16, CI 0.08, 0.41), and weaker in the high condition (β = 0.01, CI –0.09, 0.09). In addition, the moderated mediation index denoted the difference between high and low conditional effects (Index =−0.12, CI –0.22, –0.03) and the CI did not include zero. Thus, these results supported H3c.

**TABLE 7:** Results of moderated-mediation of WFF.

	WFF (M)			PWB (Y)		
Variables	β	SE	*p*	β	SE	*p*
Gender	-0.04	0.13	0.76	0.07	0.10	0.47
Age	-0.04	0.10	0.67	-0.08	0.08	0.36
Marriage	0.05	0.14	0.72	-0.14	0.11	0.20
Education	0.05	0.08	0.51	0.08	0.06	0.19
Tenure	-0.02	0.07	0.73	-0.02	0.06	0.79
PC (X)	0.57	0.08	0.00***	0.30	0.07	0.00***
POS (W)				0.20	0.08	0.01*
WFF (M)				0.14	0.05	0.006**
M*W				-0.20	0.07	0.002**
	*R*^2^ = 0.19			*R*^2^ = 0.32		
**POS**			**B**	**SE**	**LLCI**	**UICI**
-1 SD			0.16	0.04	0.08	0.41
M			0.07	0.03	0.01	0.13
+1 SD			0.01	0.05	-0.09	0.09
Index			-0.12	0.05	-0.22	-0.03

*P < 0.05, **P < 0.005, ***P < 0.001.

X, independent variable; W, moderator; M, mediator; Y, dependent variable; B, unstandardized coefficient; SE, standard error; P, probability; SD, standard deviation; LLCI, lower-level confidence interval; ULCI, upper-level confidence interval.

**FIGURE 5:**
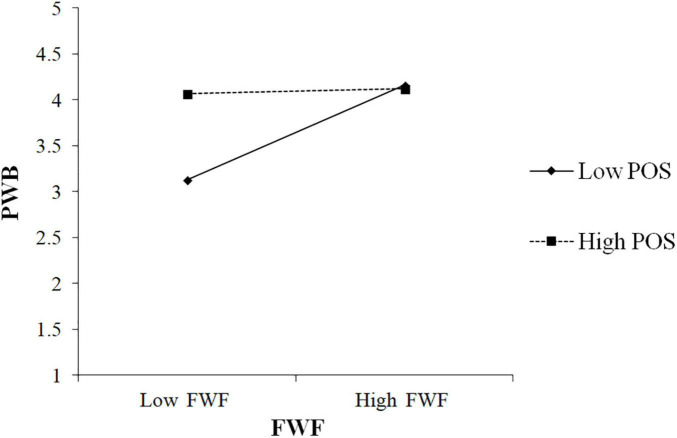
Effects of family-work facilitation (FWF) and POS on PWB.

Table 8 showed that the interaction of FWF and POS was negatively associated with PWB (β =−0.28, *p* < 0.001). To better understand the moderating effect, we followed Aiken and West (1991) to plot the interaction by calculating slopes one SD (standard deviation) above and one SD below the average value of POS. Figure 5 showed the interaction pattern. The findings in Table 8 also revealed that the conditional indirect effect was stronger in the low POS condition (β = 0.11, CI 0.05, 0.18), and weaker in the high condition (β = 0.01, CI –0.04, 0.05). In addition, the moderated mediation index denoted the difference between high and low conditional effects (Index =−0.08, CI –0.15, –0.03) and the CI did not include zero. Thus, these results supported H3d.

**TABLE 8:** Results of moderated-mediation of FWF.

	FWF (M)			PWB (Y)		
Variables	β	SE	p	β	SE	p
Gender	0.10	0.13	0.45	0.04	0.10	0.66
Age	-0.16	0.11	0.17	-0.05	0.08	0.54
Marriage	-0.21	0.14	0.13	-0.07	0.11	0.49
Education	-0.05	0.08	0.55	0.09	0.06	0.10
Tenure	0.16	0.07	0.03*	-0.06	0.06	0.31
PC (X)	0.29	0.08	0.00***	0.35	0.07	0.00***
POS (W)				0.19	0.08	0.01*
FWF (M)				0.18	0.05	0.00***
M*W				-0.28	0.07	0.00***
	*R*^2^ = 0.10			*R*^2^ = 0.35		
**POS**			**B**	**SE**	**LLCI**	**UICI**
-1 SD			0.11	0.03	0.05	0.18
M			0.04	0.02	0.01	0.08
+1 SD			0.01	0.02	-0.04	0.05
Index			-0.08	0.03	-0.15	-0.03

*P < 0.05, ***P < 0.001.

X, independent variable; W, moderator; M, mediator; Y, dependent variable; B, unstandardized coefficient; SE, standard error; P, probability; SD, standard deviation; LLCI, lower-level confidence interval; ULCI, upper-level confidence interval.

## 5. Discussion and conclusion

### 5.1. Discussion

In this study, we utilized a moderated mediation model shown in Figure 1 to present the relationship between PsyCap and PWB by incorporating two directions of conflict and facilitation and POS. Based on COR theory, this model is the first to examine the direct effect of PsyCap on PWB in the context of tourism, as well as the indirect effect of PsyCap on the abovementioned outcome through two directions of conflict and facilitation, with POS as the moderator. However, most of prior studies only focused on simple effect of PsyCap on PWB in other industries (Kim et al., 2020; Kun and Gadanecz, 2022), without in-depth exploration on underlying and conditional mechanisms behind it. Hence, there is a necessity to conduct this study.

### 5.2. Theoretical implications

We investigated the relationship between PsyCap and PWB, as well as the mediating role of WFC, FWC, WFF, and FWF and the moderating role of POS. We collected 276 valid data from tour guides in China to examine our proposed hypotheses. The results supported them and we planned to discuss our contributions based on the findings.

First, we found that PsyCap had positive influence on PWB. To the authors’ knowledge, our study is one of the first to verify the impact of PsyCap on PWB in tourism sector. An empirical study conducted by Darvishmotevali and Ali (2020) showed that PsyCap moderated the relationship between job insecurity and wellbeing. Different from our research, their study focused on the impacts of four components of PsyCap on wellbeing. However, Luthans and Youssef-Morgan (2017) have highlighted that PsyCap has a synergetic effect, which is larger than the sum of its components since it incorporates the coping mechanism that the four components have in common. Such coping mechanisms facilitate individuals’ motivation to successfully solve problems and conflicts, resulting in higher wellbeing (Choi et al., 2019).

Second, although prior studies have proved that PsyCap can predict two directions of conflict or facilitation (Karatepe and Karadas, 2014; Machín-Rincón et al., 2020; Jiao and Lee, 2021) and also foster PWB directly (Gyu Park et al., 2017; Kurt and Demirbolat, 2019), however, there is still a dearth of empirical research about the association between PsyCap and two directions of conflict and facilitation and their effects on employees’ PWB. In addition, based on the results of the current study, PsyCap had much larger indirect effect (β = 0.294, *P* < 0.01) on PWB *via* mediators than direct one (β = 0.281, *P* < 0.05). That is, the mediating role of two directions of conflict and facilitation is quite important and indispensible for the effect of PsyCap on PWB, which also verified the validity and advantages of the proposed model in this study when comparing with prior relating models.

Third, with reference to two directions of conflict or facilitation, many previous studies have reported that levels of WFC, FWC, FWF, and WFF vary with different social contexts (Ghislieri et al., 2017; Van der Lippe and Lippényi, 2020). Thus, scholars can make comparative studies on two directions of conflict or facilitation across cultures or use different cultural backgrounds as moderating variable in future hospitality and tourism studies.

Fourth, POS plays a moderating role between two directions of conflicts and facilitation and PWB. For WFC and FWC, when POS value is high, PWB is hardly affected by WFC or FWC, on the contrary, when POS value is low, PWB is significantly and negatively affected by WFC or FWC. From the perspective of WFF and FWF, when POS value is high, PWB is hardly influenced by WFF or FWF, in contrast, when POS value is low, PWB is significantly and positively influenced by WFF or FWF. That is, when the POS level is high, PWB is largely unaffected by work-family bidirectional nexuses. When the POS level is low, PWB is significantly affected by work-family bidirectional nexuses. In addition, our study confirmed that the conditional indirect influences of PsyCap on PWB through two directions of work-family conflict were stronger in the low POS condition and weaker in the high condition.

Fifth, this study expanded the COR theory. Most of existing studies have examined work-family status from the perspective of resource consumption (e.g., Hobfoll, 1989; He et al., 2019; Nauman et al., 2020). On the basis of the prior studies, we used the perspectives of resource consumption and resource supplement to examine the relationship between PsyCap, work-family status, PWB and POS. In this way, we could consider not only the stress on employees when they face a resource loss (WFC or FWC) but also the role of resource supplement when they experience (WFF or FWF).

In summary, this study breaks new ground by putting forward a new conceptual model and conducting a rigorous empirical study which links PsyCap, two directions of conflict and facilitation, PWB and POS among tour guides in China. In addition, all the hypotheses proposed in this study have been tested to be supported by the data. What’s more, all the direct, mediating and moderating effects have also been verified to be significant.

### 5.3. Practical implications

The results of the current research implied that the management of travel agencies should employ appropriate strategies to develop tour guides’ psychological resources so as to alleviate their negative work-family status and advance positive work-family status, which in turn lead to tour guides’ PWB.

First, PsyCap is considered to be malleable and can be developed within an individual (Luthans et al., 2007b). There are many face-to-face and web-based interventions which are utilized to enhance an individual’s level of PsyCap (Avey et al., 2006). This malleability is an important characteristic, because the management of travel agencies can proactively develop training programs to maintain tour guides’ PsyCap at a high level. Through training programs, tour guides’ can learn how to protect, maintain and accumulate their PsyCap more effectively (Yan et al., 2021a).

Second, if travel agencies want to advance tour guides’ PWB, they must make efforts in many aspects and attach importance to the role of tour guides’ families (Wang et al., 2021). Travel agencies should take appropriate measures to improve employees’ work-family relationship and implement family friendly policies, which can promote employees’ wellbeing to a certain extent. To be specific, family harmony and family attitude toward their careers should be taken as selection criteria when employees are interviewed. After the employee are recruited, family should be included in the team building activities or parties of the company, which can not only make the employees identify with their jobs, but also win the family’s identification with their career (Yan et al., 2022).

Finally, based on the findings that when the POS level is high, PWB is largely unaffected by work-family bidirectional nexuses. When the POS level is low, PWB is significantly affected by work-family bidirectional nexuses. Therefore, enterprises should improve POS as much as possible, because the work–family nexuses are uncontrollable factors in many cases (Wendy and Casper, 2022). Although enterprises have made a lot of efforts, the effect may not be obvious. However, POS can be improved through relevant policies of the company. When the level of POS is high enough, the relationship between work and family hardly affects the PWB of employees.

### 5.4. Limitations and future research

Although this study has made some significant contributions to existing knowledge in the field of organizational behavior, hospitality management and psychology, we must acknowledge that the current study still has some limitations which can put forward feasible prospects for future studies.

First, we employed a cross-sectional questionnaire survey process. However, cross-sectional data may have low statistical power because of its potential risk of common method bias. Therefore, longitudinal research design or sequential design can be utilized to examine the casual relationships deeply in the future. Second, only two provinces were selected, which might limit the generalizability and applicability of the conclusion in this paper. Future research can eliminate this limitation by sampling employees in different cultural regions and scenarios in different countries, and also a larger sample size is suggested. Third, this research used the item parceling technique to combine the items in the same factor and eventually synthesize a total score so as to explore the relationship between PsyCap and other variables. Given that item parceling technique may neglect some information and variance, future studies should focus more on this limitation. It is suggested to dig deeply into four components of PsyCap if they can specifically affect tour guides’ PWB.

## 6. Conclusion

Based on the COR theory, this study investigated the relationship between PsyCap, two directions of conflict and facilitation and PWB, and verified the moderating mechanism in the context of POS. The main findings of this research are as follows. First, PsyCap has a positive impact on tour guides’ PWB. Second, two directions of conflicts and facilitation could mediate the relationship between PsyCap and PWB. Third, our results show that POS not only moderates the direct influence of two directions of conflict and facilitation on PWB but also moderates the mediating role of two directions of conflict and facilitation in the relationship between PsyCap and PWB.

## Data availability statement

The raw data supporting the conclusions of this article will be made available by the authors, without undue reservation.

## Ethics statement

The studies involving human participants were reviewed and approved by the Universiti Sains Malaysia Ethics Committee and Ethical Committee of QingdaoVocational and Technical College of Hotel Management. The patients/participants provided their written informed consent to participate in this study.

## Author contributions

NB and ZY performed the conceptualization and methodology. ZY did data analysis and draft preparation. NB collected the data. RO and ZY performed the supervision. All authors listed have made a substantial, direct, and intellectual contribution to the work, and approved it for publication.
