# Supplementary material for: The moderating effect of perceived organizational support: The impact of psychological capital and bidirectional work-family nexuses on psychological wellbeing in tourism
Source: Front Psychol. 2023 Feb 23;14:1064632. doi: 10.3389/fpsyg.2023.1064632 (PMC9996001; doi:10.3389/fpsyg.2023.1064632)
Supplement: Supplementary file 1 [file Table_1.docx]

**Appendix. Measures**

| Items | Perceived organizational support (Eisenberger et al., 1986) |
| --- | --- |
| 1 | This travel agency values my contributions to its well-being. |
| 2 | This travel agency strongly considers my goals and values. |
| 3 | This travel agency is willing to help me when I need a special favor. |
| 4 | This travel agency shows very little concern for me. |
| 5 | This travel agency cares about my opinions. |
| 6 | This travel agency takes pride in my accomplishments at work. |
|  |  |
|  | Work-family conflict (Grzywacz & Marks 2000) |
| 1 | Your job reduces the effort you can give to activities at home. |
| 2 | Stress at work makes you irritable at home. |
| 3 | Your job makes you feel too tired to do the things that need attention at home. |
| 4 | Job worries or problems distract you when you are at home. |
|  |  |
|  | Family- work conflict (Grzywacz & Marks 2000) |
| 1 | Responsibilities at home reduce the effort you can devote to your job. |
| 2 | Personal or family worries and problems distract you when you are at work. |
| 3 | Activities and chores at home prevent you from getting the amount of sleep you need to do your job well. |
| 4 | Stress at home makes irritable at work. |
|  |  |
|  | Work-family facilitation (Grzywacz & Marks 2000) |
| 1 | The things you do at work help you deal with personal and practical issues at home. |
| 2 | The things you do at work make you a more interesting person at home. |
| 3 | Having a good day on your job makes you a better companion when you get home. |
| 4 | The skills you use on your job are useful for things you have to do at home. |
|  |  |
|  | Family- work facilitation (Grzywacz & Marks 2000) |
| 1 | Talking with someone at home helps you deal with problems at work. |
| 2 | Providing for what is needed at home makes you work harder at your job. |
| 3 | The love and respect you get at home makes you feel confident about yourself at work. |
| 4 | Your home life helps you relax and feel ready for the next day’s work. |
|  |  |
|  | Psychological well-being (Grossi et al., 2006) |
| 1 | Have you been bothered by nervousness or your "nerves" during the past month? |
| 2 | How much energy, pep, or vitality did you have or feel during the past month? |
| 3 | I felt downhearted and blue during the past month. |
| 4 | I was emotionally stable and sure of myself during the past month. |
| 5 | I felt cheerful, lighthearted during the past month. |
| 6 | I felt tired, worn out, used up, or exhausted during the past month. |
|  |  |
|  | Psychological capital (Luthans et al., 2007) |
| 1 | I have a good sense of why I have certain feelings most of the time. |
| 2 | I have good understanding of my own emotions. |
| 3 | I really understand what I feel. |

*According to copyrights terms of PCQ-24, it is not allowed to present an entire instrument in a publication, thus, three sample items are shown above.*
